# Atomic Model and Micelle Dynamics of QS-21 Saponin

**DOI:** 10.3390/molecules19033744

**Published:** 2014-03-24

**Authors:** Conrado Pedebos, Laércio Pol-Fachin, Ramon Pons, Cilâine V. Teixeira, Hugo Verli

**Affiliations:** 1 Centro de Biotecnologia, Universidade Federal do Rio Grande do Sul, Av Bento Gonçalves 9500, CP 15005, Porto Alegre 91500-970, RS, Brazil; E-Mails: conrado.pedebos@ufrgs.br (C.P.); laerciofachin@cbiot.ufrgs.br (L.P.-F.); 2 Departamento de Química Fundamental, Universidade Federal de Pernambuco, Av Prof. Luiz Freire s/n, Cidade Universitária, Recife 50740-540, PE, Brazil; 3 Departament de Tecnologia Química i de Tensioactius, Institut de Química Avançada de Catalunya, IQAC-CSIC, Jordi Girona, 18-26, Barcelona 08034, Spain; E-Mail: ramon.pons@iqac.csic.es; 4 Instituto de Física, Universidade Federal do Rio Grande do Sul, CP15051, Porto Alegre 91501-970, RS, Brazil; 5 Unitat de Biofísica, Facultad de Medicina, Universitat Autonoma de Barcelona, Cerdanyola del Vallès 08193, Spain

**Keywords:** QS-21, saponins, glycoconjugates, molecular dynamics, SAXS, micelle, adjuvant

## Abstract

QS-21 is a saponin extracted from *Quillaja saponaria*, widely investigated as a vaccine immunoadjuvant. However, QS-21 use is mainly limited by its chemical instability, significant variety in molecular composition and low tolerance dose in mammals. Also, this compound tends to form micelles in a concentration-dependent manner. Here, we aimed to characterize its conformation and the process of micelle formation, both experimentally and computationally. Therefore, molecular dynamics (MD) simulations were performed in systems containing different numbers of QS-21 molecules in aqueous solution, in order to evaluate the spontaneous micelle formation. The applied methodology allowed the generation of micelles whose sizes were shown to be in high agreement with small-angle X-ray scattering (SAXS). Furthermore, the ester linkage between fucose and acyl chain was less solvated in the micellar form, suggesting a reduction in hydrolysis. This is the first atomistic interpretation of previous experimental data, the first micellar characterization of saponin micelles by SAXS and first tridimensional model of a micelle constituted of saponins, contributing to the understanding of the molecular basis of these compounds.

## 1. Introduction

**Figure 1:**
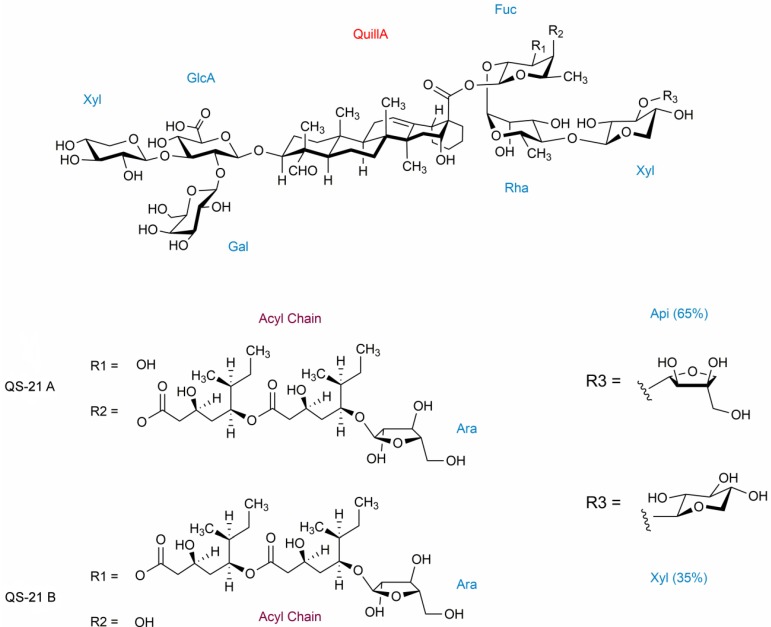
QS-21 molecular structure and its heterogeneity. The carbohydrates (blue) are distributed as a branched portion and a linear portion. Quillaic acid (QuillA—red) and the acyl chain (purple) make up the hydrophobic units of the compound.

QS-21 is a saponin extracted from the bark of the *Quillaja saponaria* tree, well-known by its role as an immunostimulating compound, being one of the main targets of a great number of clinical trials [1] and synthesis of variants [2,3,4]. Its structure (Figure 1) is composed of two different carbohydrate chains (one linear and one branched) with the addition of an acyl chain [5].

This saponin presents two regioisomers in solution, QS-21 A and QS-21 B, in a proportion of 20:1, respectively, with QS-21 A being more thermodynamically stable [5,6]. The sole difference between these isomers is the position of the ester linkage that links the acyl chain moiety and the fucose residue composing its structure [5]. Moreover, distributed among these regioisomers, there is sugar heterogeneity (Figure 1) at the R_3_ fragment of the saponin’s structure, involving the presence of an apiose (65%) or a xylose (35%) sugar residue. Consequently, in aqueous media four different compounds exist: QS-21 A-apio, QS-21 A-xylo, QS-21 B-apio and QS-21 B-xylo.

The acyl chain is believed to play an important role in a higher extent to Th1 response and in a lesser extent to Th2 type responses [7] promoted by the use of QS-21 as an adjuvant. This portion of the saponin’s structure is also a site of degradation by hydrolysis at the ester linkage, a feature that is responsible for the decrease of the shelf life of this compound [6] and the action in Th1 activity and cytotoxic T lymphocytes production [8]. Another property of these molecules involves their behavior in water: as saponins are constituted by a hydrophobic (the triterpene ring) and a hydrophilic part (sugar groups), they form micelles in aqueous solution at a critical micellar concentration (cmc) around 0.5 g/L. This concentration can vary, depending on the solution conditions (pH, temperature, salt addition, presence of cholesterol), reaching a c.m.c of 51 μg/mL [9] in succinate buffered aqueous solution. An additional important property of QS is its interaction with cholesterol, which, besides making it promising for the food industry, is explored for the preparation of Immune Stimulating Complexes (ISCOMs), which are used for delivery of vaccine antigens, targeting the immune system [10]. Actually, the adjuvant properties of QS-21 have been observed even at concentrations as low as 20 μg/mL [11], at which only monomers are found in solution. This indicates that the formation of micelles is not essential to its immunopotentiator activity [9]. However, it is suggested that, when aggregated in a micelle, the QS-21 saponins tend to bury their acyl chains due to its hydrophobic nature, generating an apolar interior for the aggregates, which is thought to give stability to QS-21 [6]. Furthermore, both its immunoadjuvant activity and its toxic or non-toxic effects are directly related to its interaction with membranes which, as well as its interaction with cholesterol, is directly related to its amphiphilic properties. Therefore, a proper knowledge of dynamic micellar behaviour of the QS-21 in aqueous solution is very important to elucidate its biological and toxic activities.

Structure-based investigations can help understand the molecular basis concerning these compounds’ dynamics [12], conformation [13], and function [14]. However, obtaining 3D atomic models remains a challenge, since efficient approaches that accurately describe the biological conformational states of saponins and glycoconjugates are still scarce. Considering that the understanding of the molecular behavior of QS-21 may support further studies involving the development of immunopotentiators, we aimed to characterize the conformational ensemble of the saponins QS-21 A and B (both as the -apio major isomer), as well as examine the spontaneous formation of micelles composed by these molecules, both experimentally and theoretically. For the theoretical characterization of the saponin we used a previously applied approach [15] involving molecular dynamics (MD) simulations with compounds of the same kind. The experimental study was carried out by small-angle X-ray scattering (SAXS). The two methods were performed independently and we were able to compare the results and further validate the models constructed. Thus, we characterized QS-21 micelles by SAXS for the first time and provided an atomic 3D model for this saponin and the micelle aggregation of this compound, constituting the first atomic level detailed evidence for the micelle formation.

## 2. Results and Discussion

### 2.1. Titration by Fluorescence

**Figure 2:**
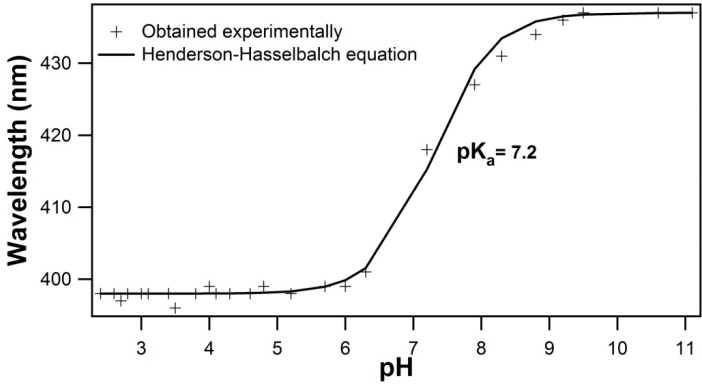
Titration curve of QS-21 in aqueous solution.

Due to the presence of the carboxyl group, which can be negatively charged or neutral depending on the pH, we performed a titration in order to determine the pKa of QS. Figure 2 shows the emission wavelength of QS as a function of pH, whose fitting of Henderson-Hasselbalch equation gives a pKa value of 7.2 ± 0.1. Thus, the samples for SAXS measurements were prepared at pH 2, so that we are sure that the micelles have no net charge.

### 2.2. Micellar Structure by SAXS

**Figure 3:**
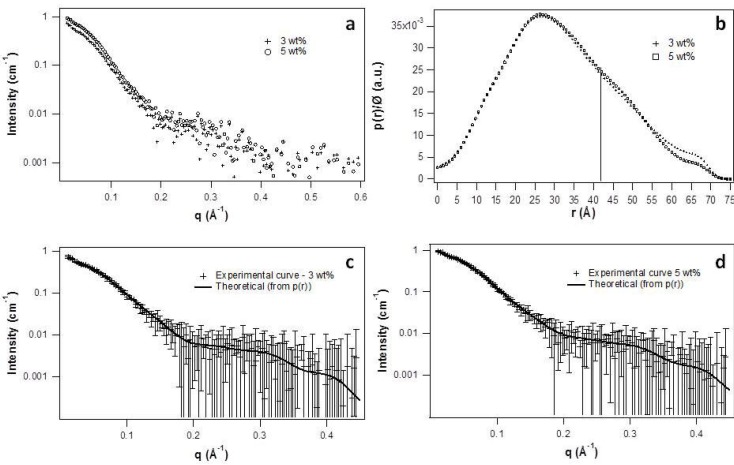
(**a**) SAXS curves; (**b**) Distance distribution function of QS in aqueous solution at 3 and 5 wt%; Theoretical intensity, calculated from the p(r) functions, together with the experimental curve for (**c**) 3 wt% and (**d**) 5 wt%.

The scattering curves of the samples containing 3 wt% and 5 wt% of QS in solution are shown in Figure 3a, from which the distance distribution function was calculated. Interference between micelles shows up at small q-values. For this reason, points at the beginning of the curve were removed for the calculation of p(r), so that obtained p(r) values were due only to the form factor of the micelles and converged smoothly to zero as r reached D_max_ (the biggest dimension of the particles). The p(r) curves obtained for both concentrations, normalized with the QS volume fraction, are shown in Figure 3b. The same D_max_ value of 75 ± 3 Å was obtained for both concentrations, indicating that the micelles size remains constant. Indeed, both p(r) curves are identical even at small-r values, which means that they have the same scattering length profile and the same cross section radius [16]. The theoretical scattering curves calculated from the obtained p(r) functions are plotted together with the experimental curves, showing a good agreement. It can be noted that the p(r) curves are slightly asymmetric towards higher r-values, which indicates that the particles are not perfectly spherical, but elongated. When the length of the particles is much longer than their cross section, a linear decay appears at the p(r) curve, and the point of inflection between the maximum of the function and the linear region of the curve gives a rough estimation of the cross section size [17]. In the present case, the linear region is not clearly defined, indicating that the axial ratio between the axis and the cross section is not high, although we can identify the inflection point at around 42 Å. The equivalent axial ratio is 1.8 ± 0.2.

### 2.3. QS-21 Conformational Characterization

**Figure 4:**
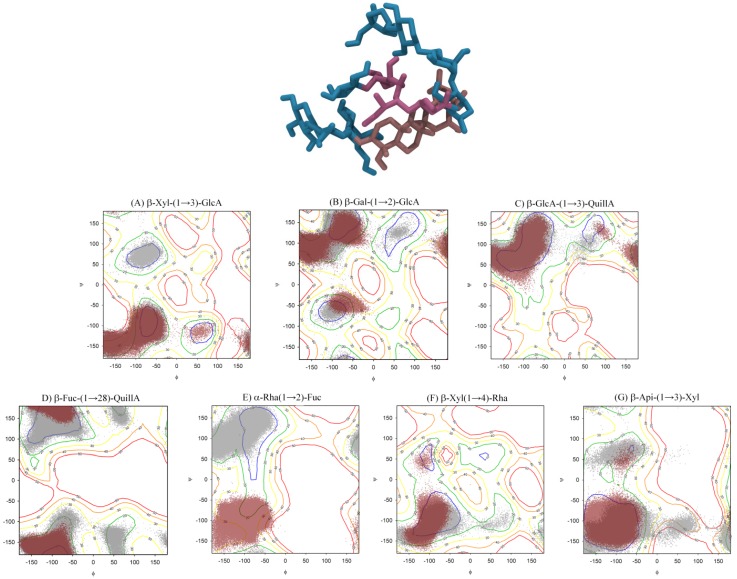
(**Top**) Representation of the whole molecule of QS-21 in its most prevalent conformation: the brown and purple parts represent the hydrophobic region and the blue part its hydrophilic portion. (**Bottom**) Energy contour plots for the glycosidic linkages that compose the QS-21 saponin. The corresponding aqueous solution populated geometries are superimposed as isolated units (gray dots) or as the complete saponin structure (dark red dots). Contour levels are shown at every 10 kJ·mol^−1^, from 10 to 50 kJ·mol^−1^.

To properly describe the conformational ensemble adopted by QS-21 saponin in solution, we initially evaluated the glycosidic linkages that compose this molecule by employing energy contour plots (Figure 4). The glycosidic linkages are identified as β-d-Xyl-(1→3)-GlcA, β-d-Gal-(1→2)-GlcA, β-d-GlcA-(1→3)-QuillA, β-d-Fuc-(1→28)-QuillA, α-l-Rha-(1→2)-Fuc, β-d-Xyl-(1→4)-Rha and β-d-Api-(1→3)-Xyl. Since the two linkages involving Fuc and the Acyl Chain, as well as between the latter and Ara cannot be considered as glycosidic, we included the calculations of atomic charges and the conformation of these structures in aqueous solution, as obtained by MD (described in the Experimental Section).

We performed a comparison between the three states presented in the energy contour plots, that is, vacuum, isolated disaccharide in solution and complete saponin in solution. Through these plots, the minimum energy geometries of each bond, the co-existence of multiple conformations and its most populated angles were evaluated. The disaccharidic units in solution populated in a similar manner to the vacuum profile, reinforcing the role of the glycosidic linkage pattern over their conformation determination, as previously reported [12]. The geometries populated when the saponin was fully assembled presented only one conformational change: the ψ angle from the α-l-Rha-(1→2)-Fuc linkage (Figure 4E), which shifted from a positive value of around 120° to a negative value of −120°. A possible explanation for this shift is that the hydrophilic portions of the molecule enclose the hydrophobic core, which contains the Acyl Chain and QuillA, reducing the solvent accessible area and stabilizing this new conformation. Still, the mere presence of the Acyl Chain might be sufficient to influence the adjacent monosaccharide units. Besides, the disaccharidic units presented a larger number of populated conformational states when compared to the complete model angles, in agreement with previous reports [12].

### 2.4. Micelle Formation and Dimensions

**Figure 5:**
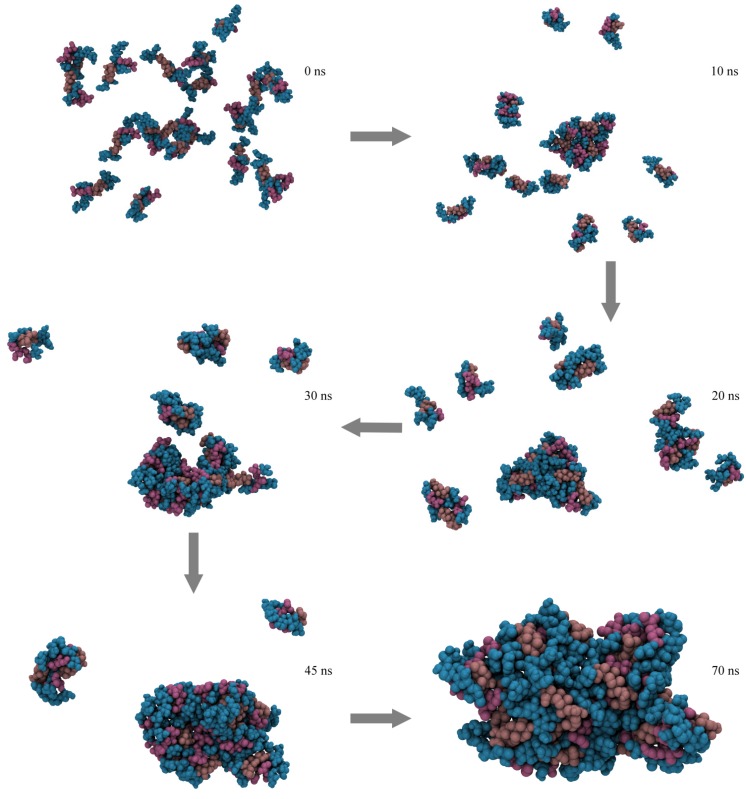
Assembly of a micelle composed by 20 molecules of QS-21 after a 0.1 μs MD simulation. The carbohydrates are represented in blue, while QuillA and the acyl chains are represented in brown and purple, respectively.

Subsequently to the MD simulations performed to obtain an initial model for QS-21, in its glycosidic linkages optimal conformational states, we employed the fully assembled saponin in new μs time scale MD simulations, aiming to observe spontaneous aggregation of these compounds. Systems were composed by 1, 2, 3, 4, 10 and 20 molecules of QS-21 A and B, which were randomly inserted in the simulation box. In all systems, the saponins have aggregated in a gradual manner, spontaneously forming micelles in varying time frames. These micelles, however, are not assembled in a traditional fashion, but rather in a disorganized one (Figure 5), where the multiple portions of the saponin are freely interacting with each other.

**Figure 6:**
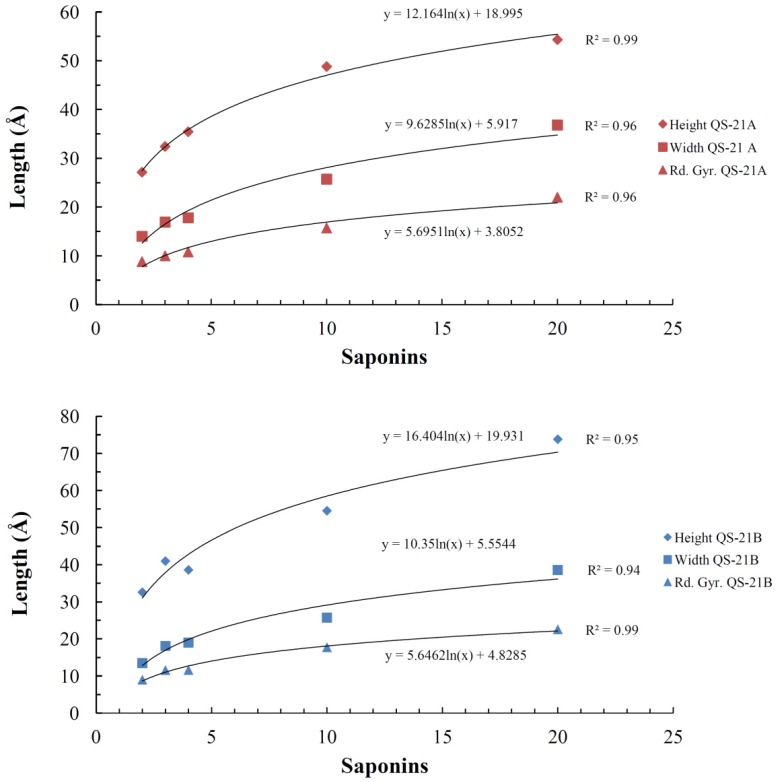
Average dimensions for each micelle pertaining to the independent simulations of QS-21 A and QS-21 B.

**Figure 7:**
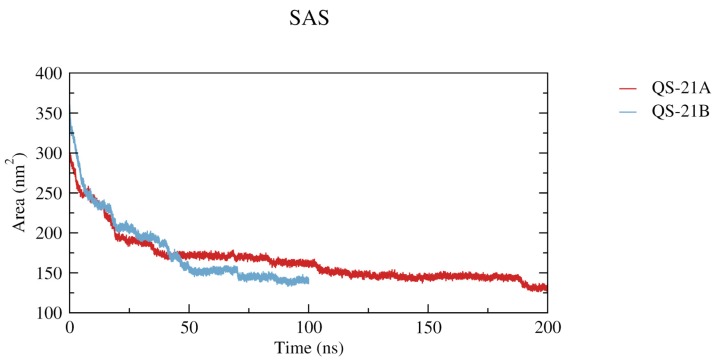
Solvent accessible surfaces for (QS-21A)_20_ and (QS-21B)_20_ micelles. When equilibrated, (QS-21A)_20_ presents an average value of 131.8 ± 2.1 nm^2^, while (QS-21B)_20_ presents an average of 139.9 ± 2.0 nm^2^.

To evaluate the micelle formation, we calculated the radius of gyration and the dimensions (X and Y) of each final aggregate (Table 1). In general, most micelles dimensions have shown increasing values as the number of saponins in the systems also raised (Figure 6). All calculations performed led to the formation of micelles spontaneously, despite occurring at different time scales, as indicated by the solvent accessible surface graphics (Figure 7—QS-21A = 0.2 μs and QS-21B = 0.1 μs).

**Table 1:** Dimensions (radius of gyration, height and width) of the assembled micelles for each MD simulation of QS-21A and QS-21B. All values are given in Å.

System (QS-21 molecules)	Saponin							
	QS-21A				QS-21B			
	Rg	Height	Width	Axial Ratio	Rg	Height	Width	Axial Ratio
2	8.8 ± 0.5	27.1 ± 2.1	14.0 ± 1.0	1.9 ± 0.2	9.0 ± 0.5	32.6 ± 0.5	13.5 ± 1.0	2.4 ± 0.2
3	10.0 ± 0.2	32.4 ± 1.4	16.9 ± 0.4	1.9 ± 0.1	11.6 ± 0.2	41.0 ± 0.2	18.1 ± 0.2	2.3 ± 0.1
4	10.8 ± 0.2	35.4 ± 1.9	17.8 ± 0.7	2.0 ± 0.1	11.6 ± 0.3	37.2 ± 0.3	19.0 ± 0.8	2.00 ± 0.03
10	15.7 ± 0.1	48.8 ± 1.6	25.7 ± 0.8	1.9 ± 0.1	17.8 ± 0.3	54.5 ± 0.3	25.7 ± 1.2	2.1 ± 0.1
20	22.0 ± 1.4	54.3 ± 1.1	36.8 ± 1.6	1.5 ± 0.1	22.0 ± 1.3	72.0 ± 1.2	39.2 ± 1.1	1.8 ± 0.1

We applied logarithmic trend lines in every graphic of each property (Figure 6—height, width and radius of gyration) and were able to obtain a high correlation R value for all the curves. This suggests that this process occurs in a progressive manner until reaching a plateau, where new micelles would be formed, instead of aggregating to the main one. Moreover, based on the obtained data and its high correlation (Figure 6), we may attempt to predict the approximate dimensions for any size of micelles.

### 2.5. Ester Linkage Solvation

**Figure 8:**
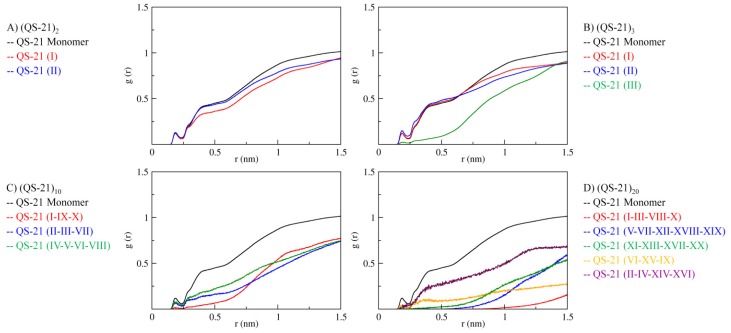
Radial distribution function of the ester linkage between Fuc and Acyl. (**A**) MD simulation of two saponins (QS-21)_2_; (**B**) MD simulation of three saponins (QS-21)_3_; (**C**) MD simulation of ten saponins (QS-21)_10_; (**D**) MD simulation of twenty saponins (QS-21)_20_. A comparison with a monomeric QS-21 (black) was performed in all graphics. In C e D, some lines represent an approximation between similar results, so that the roman numerals indicate which saponins are included in the respective line.

An aspect involving the micelles composed by saponin QS-21 is the diminished degradation of the acyl chain when compared to the monomeric form, increasing its shelf life [6]. Accordingly, we performed a Radial Distribution Function (Figure 8) searching for solvent molecules that were near the atoms constituting the ester bond between Fuc and the acyl chain. In the comparison, we used the monomeric QS-21 as a reference and all the systems simulated independently. System (QS-21)_2_presents a slight decrease in one of the curves (QS-21 (I)), whereas in system (QS-21)_3_, the (QS-21 (III)) curve has almost no solvent molecules in the nearest hydration layers. In systems (QS-21)_10_ and (QS-21)_20_, both simulations presented major decrease on more than one curve, containing groups of saponins. The RDF analysis demonstrated a reduction in the accessibility of the aqueous solvent to the ester linkage as the number of molecules increase, identifying that, when organized in micelles, QS-21 saponins may present an increased protection against degradation by spontaneous hydrolysis.

### 2.6. Comparison of Both Methods in the Micelle Structure Analysis

**Figure 9:**
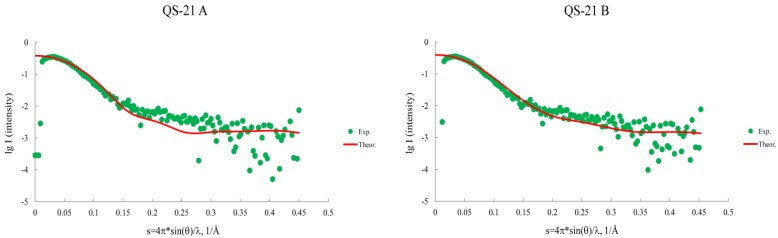
Comparison between data obtained by computational simulations and SAXS. The experimental curve (green symbols) is fitted to the theoretical curve (red line), using CRYSOL server and the final micelle model obtained in the simulation.

We compared the results obtained by SAXS to the final structure obtained in the 20 molecules system, since its dimensions were the closest to the obtained experimentally. For such a comparison, we fitted (Figure 9) the experimental curve (SAXS) to the theoretical curve (simulation) and observed its superimposition. Additionally, the experimental SAXS dimensions data could be also compared to the simulations (Table 2).

**Table 2:** Comparison between the dimensions (radius of gyration, height and width) obtained by MD simulations of (QS-21A)_20_ and (QS-21B)_20_ and by SAXS experiments. All values are given in Å.

System	Saponin						SAXS		
	QS-21A			QS-21B					
	Rg	Height	Width	Rg	Height	Width	Rg	Height	Width
20	22.0 ± 1.4	54.3 ± 1.1	36.8 ± 1.6	22.6 ± 2.1	73.8 ± 2.9	38.6 ± 1.7	25.0 ± 3.9	75.0 ± 3.0	42.0 ± 3.0

Data obtained by the CRYSOL server [18] for RGt (QS-21 A = 20.28 Å and QS-21 B = 21.23 Å) are in close agreement with the RGe (QS-21 A = 22.00 Å and QS-21 B = 22.6 Å), as well as the correlation of the curves as observed in the graphic. The Angular Units equation selected was s = 4πsin(θ)/λ, 1/Å, and the remaining options were set to default. The *chi* values, which indicate the discrepancy between the theoretical and experimental curves, are 10.0 for QS-21A data and 9.5 for QS-21B data. The (QS-21A)_20_ system appears to be a more compact aggregate, since it has the same number of saponins as (QS-21B)_20_, but lower dimensions. Hence, a greater number of molecules would be required in order to achieve the experimental dimensions. The dimensions obtained for (QS-21B)_20_ are in closer agreement to what has been found by SAXS, although the axial ratio obtained by the simulations (Table 1) and the SAXS experiments (1.8 ± 0.2) is in good agreement for both micelles (20 saponins). This may indicate that MD simulations can properly describe the aggregation process involving QS-21 saponins which can be compared with experimental data, such as SAXS.

## 3. Experimental

### 3.1. Saponin Preparation

Saponin from Quillaja bark (S7900 from Sigma, Madrid, Spain) was dissolved in hot water and extracted with butanol, which was then evaporated. The resulting paste was redissolved in methanol. The methanol was evaporated and the sample was further lyophilized. The obtained powder was then mixed in aqueous solution at the desired concentration.

### 3.2. Titration

The pKa of the QS in water was determined by fluorescence, for a concentration of 0.1 g/L, by varying the pH from 2.4 to 11.1. The measurements were carried out in a SLM-Aminco 8000 spectrophotometer (SLM Instruments, Urbana, IL, USA), at room temperature. Excitation wavelength was set to 298 nm and emission scans were performed from 330 to 550 nm.

### 3.3. Small-Angle X-ray Scattering (SAXS)

Water solution with HCl at pH 2 containing 3 wt% and 5 wt% of QS were prepared and measured in a glass capillary of 1.0 mm inner diameter. The measurements were performed in a Kratky compact camera (Hecus X-ray systems, Graz, Austria) coupled to a Siemens KF 760 (3 kW) generator, at room temperature (298 K). A Ni-filter was used to obtain a CuK_α_ radiation (1.542 Å). Slits were used for collimation producing a line beam. A linear position sensitive detector (PSD-OED 50 M-Braun, Graz, Austria) was used. The samples were measured for 1 h. The scattering curves were smoothed by fitting a third-degree polynomial to adjacent points and then desmeared by Singh procedure [19]. Then they were corrected for the solvent scattering and put in an absolute scale by using the transmission value obtained with a moving slit device and the standard value of the scattering of water (1.68 × 10^−2^ cm^−2^) [20,21]. The scattering intensity is given as a function of the scattering vector:

*q* = 4*π*sin *θ* / *λ* (1)

where λ is the wavelength and 2θ is the scattering angle. *q* ranged from 0.02 to 0.6 Å^−1^.

### 3.4. Analysis Method

As this is the first SAXS study of QS-21 micelles, there was no a priori information on the system. For this reason we used a model-independent approach, which calculates the pair-distance-distribution function, p(r), as the Fourier Transform of the measured intensity. This function gives an evaluation of the overall symmetry and dimension of the particles. The p(r) function is related to the scattering intensity by Glatter [17]:

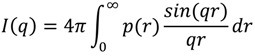
(2)

As the scattering curve is measured only in a finite-q range, we cannot perform a traditional Fourier Transformation. This problem is overcome by using the Indirect Fourier Transformation method, developed by Glatter [22]. In this method, the integral can be performed up to a value D_max_, which is the maximum dimension of the micelle. The pair-distance distribution function is given by a linear combination of a finite number N of cubic *B*-spline functions, ϕ_i_(r)

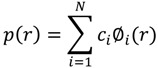
(3)
where c_i_ is the coefficient of the ∅_i_ function. Inserting (3) in (2) and using a least-squares method together with a stabilization routine, the coefficients c_i_ can be determined. When r ≥ D_max_, the p(r) function is zero. The scattering length density of the particles can also be calculated by the square-root deconvolution, using a similar method to the one described above [23]. The scattering length function can be calculated either as a smooth spline curve or as a step function. This procedure was carried out by using a package developed by Glatter.

### 3.5. Nomenclature, Topologies, and Software

The IUPAC recommendations and symbols of nomenclature [24] were adopted. Two contiguous carbohydrate residues, or a monosaccharide and QuillA, had their orientation described appropriately by evaluating their glycosidic linkage torsional angles. For a (1→X) linkage, where “X” is “2”, “3”, “4” or “28” for the (1→2), (1→3), (1→4) or (1→28), respectively, the φ and ψ dihedral angles are defined as shown in (4) and (5):

φ = O5–C1–O1–CX(4)

ψ = C1–O1–CX–C(X − 1)(5)

For a (1→28) linkage, Omega (ω) is defined as below:

ω= O28A–C28–C17–C16(6)

The topologies for saccharides, quillaic acid (QuillA) and acyl chain have been generated by the PRODRG server [25]. Structures were manipulated using PyMOL [26], VMD [27,28] and MOLDEN [29]. The MD simulations and analyses were performed using GROMACS simulation suite, version 4.0.5 [30], and GROMOS96 43a1 force field [31].

### 3.6. Saponin Construction and Topologies Refinement

The building block methodology [32] was applied, aiming to obtain the starting structures for the QS-21 conformational study. The compound was constructed using its most prevalent conformations of its minimal components in solution (disaccharides and linkages between a monosaccharide and QuillA or a monosaccharide and an acyl chain). Indeed, all of such units were constructed with the MOLDEN software and submitted to PRODRG server to obtain their crude topologies and atomic coordinates. Refinements were added on these topologies, including HF/6-31**-derived Löwdin atomic charges, as obtained from previous works [15,33], or calculated with the GAMESS software [34], in the case of the carboxyl group from GlcA (protonated) and acyl chain atomic charges. Improper dihedrals were added to maintain the conformational states ^1^C_4_ for the α-l-rhamnose (α-l-Rha), ^4^C_1_ for β-d-xylose (β-d-Xyl), β-d-galactose (β-d-Gal), β-d-fucose (β-d-Fuc), and β-d-glucuronate (β-d-GlcA), ^4^T_3_ for the α-l-arabinose (α-l-Ara), and E_3_ for β-d-apiose (β-d-Api) residues. Also, proper dihedrals were included, as described in GROMOS96 force field. Aiming to obtain a starting geometry for the Fuc—Acyl Chain—Ara unit, we performed a MD simulation of 0.1 μs of such structure in aqueous solution. Subsequently, the entire trajectory was clusterized (employing the *g_cluster* tool from GROMACS package) in order to collect the most prevalent structures.

### 3.7. Contour Plots

The disaccharidic or monosacharide-aglycone glycosydic linkages presented by QS-21 had their torsion angles rotated between −180° and 150°, in steps of 30°, thus generating 144 conformers for each torsion, allowing us to obtain a conformational description of the molecule. We used a constant restriction force in the φ and ψ proper dihedrals in this energy minimization process, which permitted the exploration of the conformational space by all of the unit’s linkages [13]. Thus, these minimized conformations were submitted to MD simulations in vacuum for 20 ps at 10 K, with an integration step of 0.5 fs, further improving the minimum-energy conformations investigation. The identified low energy conformations in this procedure were employed as starting geometries for 0.1 μs MD simulations in aqueous solution and 298 K. This procedure allowed an enhanced sampling of the glycosydic linkages dihedral angles of the units.

### 3.8. MD Simulations

MD simulations were performed in 0.1 and 0.2 μs scale aqueous solutions (SPC water model) [35], inside a solvated cubic box and making use of periodic boundary conditions. To best resemble the experimental environment, we respected the 3 wt% concentration in each simulation, obtaining systems that presented up to ~220,000 atoms (20 saponins in water). The covalent bond lengths were constrained by the LINCS method [36], so that an integration step of 2 fs was applied after an energy minimization with Steepest Descents algorithm. For the calculation of the electrostatic interactions, we employed the Particle Mesh Ewald method [37]. The temperature (298 K) and pressure (1 atm) of the saponins and solvent were also kept constant, by applying external temperature and pressure baths with coupling constants of τ = 0.1 and 0.5 ps [38], respectively. The V-rescale thermostat and the Berendsen barostat were employed in the simulations. Cutoff values were set to 0.9 nm for short-range interactions. The van der Waals interactions were truncated at 0.9 nm. The GROMACS package analysis tools were employed in many analyses: the *g_gyrate* tool was employed to calculate the radius of gyration of the micelles, while the *g_dist* and *g_mindist* tool were used to calculate their dimensions and the *g_rdf* tool was applied to obtain the Radial Distribution Function. We collected the last 15 ns of the (QS-21A)_20_ and (QS-21B)_20_ micelles simulation, since the full assembly of these structures occurred at 185 ns in the (QS-21A)_20_ simulation and at 70 ns in the (QS-21B)_20_ simulation.

## 4. Conclusions

For the first time, the structure and conformation of QS-21 in micelles were determined. A 3D atomic model was built by molecular dynamics, which showed that the higher the number of QS-21 molecules in a micelle, the lower the accessibility of the aqueous solvent to ester bond between Fuc and the acyl chain, feature that is related to the storage stability. The characterization of the conformational ensemble of this glycoconjugates is not a simple task, due to its high flexibility and difficulties regarding crystallization, and usually demands the use of other experimental methods, such as NMR [39]. We have been employing MD simulations as a tool to successfully describe the conformational profiles that occur in carbohydrates [12] and glycoconjugates [15].

The presence of so many groups in the QS-21 molecule makes the study of micelles by SAXS a very difficult accomplishment. To the best of our knowledge, this is the first report on QS-21 micelles by SAXS. The combination of SAXS and MD simulations allowed us to understand the molecular conformation of QS-21 in the micelles, as well as the aggregation process. The present results and methods can be employed in further studies to investigate mechanisms of immunostimulation in which this compound is involved, that are so far unknown. Furthermore, we were able to observe the protection of the acyl chains against solvent molecules by analyzing the ester linkage between acyl moieties and the Fuc residue.
